# Nanocarrier-mediated foliar zinc fertilization influences expression of metal homeostasis related genes in flag leaves and enhances gluten content in durum wheat

**DOI:** 10.1371/journal.pone.0191035

**Published:** 2018-01-17

**Authors:** Paresh Deshpande, Ashwin Dapkekar, Manoj Oak, Kishore Paknikar, Jyutika Rajwade

**Affiliations:** 1 Nanobioscience group, Agharkar Research Institute, Pune, India; 2 Savitribai Phule Pune University, Ganeshkhind, Pune, India; 3 Genetics and plant breeding, Agharkar Research Institute, Pune, India; National Institute of Plant Genome Research, INDIA

## Abstract

**Background:**

Wheat is the staple food for most of the world’s population; however, it is a poor source of zinc. Foliar fertilization of zinc via zinc loaded chitosan nanocarriers (Zn-CNP) post-anthesis has proved to be a promising approach for grain zinc enhancement in durum wheat as evidenced in our earlier study. However, the molecular mechanism of uptake of zinc via Zn-CNP remains unclear.

**Methods/Principle findings:**

Foliar application of Zn-CNP was performed at post anthesis stages in two durum wheat cultivars (MACS 3125 and UC1114, containing the *Gpc-B1* gene), and expression levels of several metal-related genes were analyzed during early senescence. Zn-CNP application indeed caused changes in gene expression as revealed by qPCR data on representative genes involved in metal homeostasis, phloem transporters, and leaf senescence. Furthermore, zinc-regulated transporters and iron (Fe)-regulated transporter-like protein (ZIP) family [*ZIP1*, *ZIP7*, *ZIP15*], *CA* (carbonic anhydrase), and *DMAS* (2’-deoxymugineic acid synthase) in flag leaves exhibited significant correlation with zinc content in the seeds. The analysis of grain endosperm proteins showed enhancement of gamma gliadins while other gluten subunits decreased. Gene expression within ZIP family members varied with the type of cultivar mostly attributed to the *Gpc-B1*, concentration of external zinc ions as well as the type of tissue analyzed. Correlation analysis revealed the involvement of the selected genes in zinc enhancement.

**Conclusion:**

At the molecular level, uptake of zinc via Zn-CNP nanocarrier was comparable to the uptake of zinc via common zinc fertilizers i.e. ZnSO_4_.

## Introduction

Zinc (Zn) is an essential micronutrient required for all living organisms. Zinc plays an important role in the normal physiological process of growth and development [1]. It acts as a cofactor for more than 300 enzymes that are involved in different biological processes which include DNA replication, protein, and lipid metabolism and in gene regulation (zinc fingers transcription factors) [2, 3]. Deficiency of this micronutrient causes growth retardation, cognitive impairment, immune dysfunction, mental lethargy, skin related disorders, delayed wound healing, diarrhea in children etc. [4, 5]. It is reported that more than 50% of the world population is zinc deficient and more than 80% of the South Asian population is at a risk of zinc deficiency [6, 7]. Zinc deficiency is prevalent in vegetarians consuming mostly cereal-based foods, supplemented with very little vegetables, fruits and nuts, and containing anti-nutrients. Cereals are inherently low with respect to their zinc content, which is a primary cause of zinc deficiency. Therefore, increasing the zinc content of staple cereals to reduce the zinc deficiency is recommended [8].

**Fig 1:**
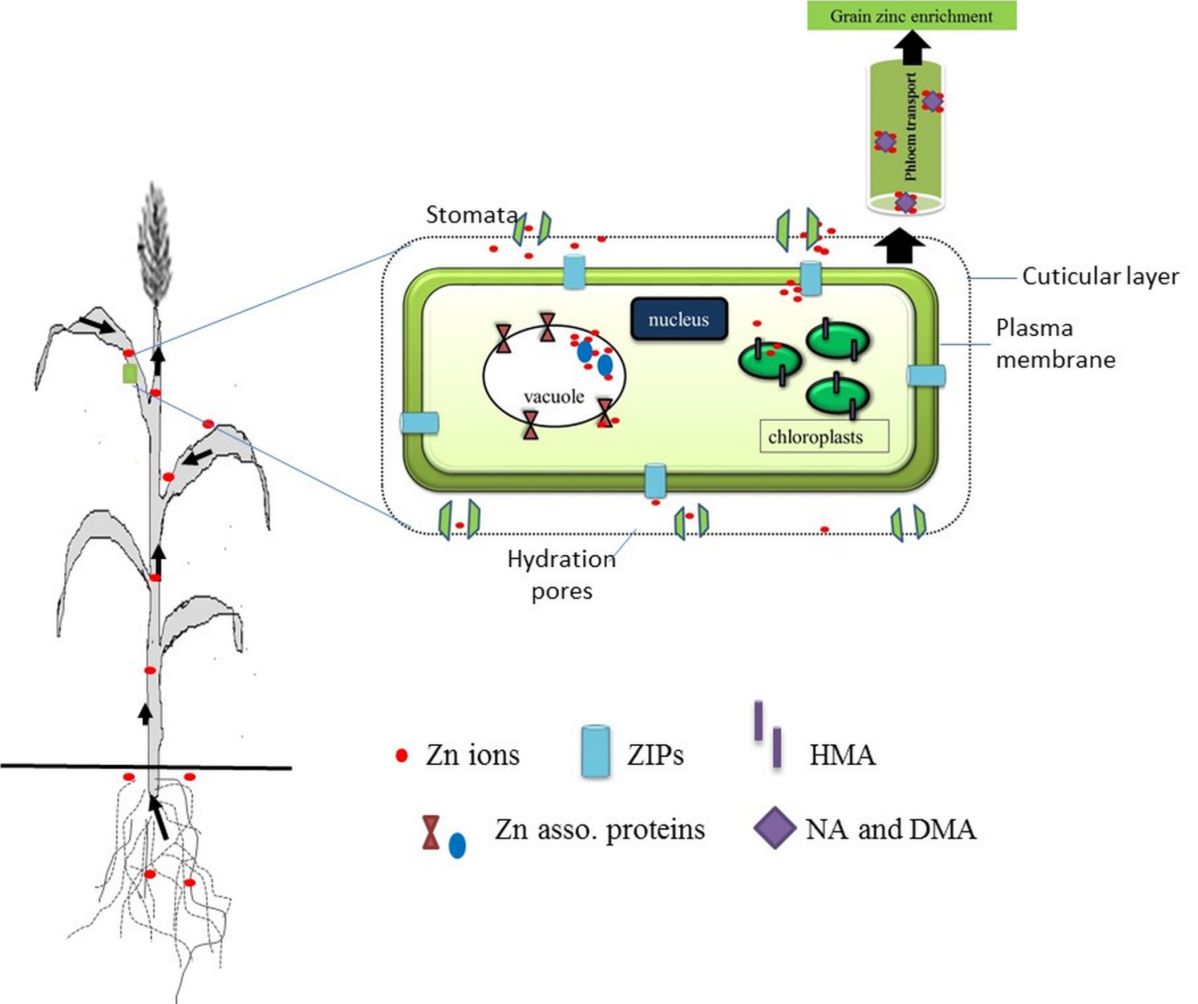
The physiological process of zinc uptake in a wheat plant via roots and leaves.

To mitigate the zinc deficiency through biofortification of commonly consumed cereals viz., rice, wheat, maize, sorghum etc. several national and international programs are initiated. Biofortification can be achieved both, by genetic modification and breeding for crops with better micronutrient uptake traits and by following agronomic practices which mainly focus on nutrient fertilization. In the latter, especially with reference to zinc insufficiency, micronutrient application by foliar route is an ideal short-term remedy for increasing the zinc content in the edible portion of the plant i.e. grains. [9, 10] Foliar fertilization particularly during the grain development stage in the life cycle of the plant leads to high grain zinc concentrations [11, 12]. The physiological process of zinc uptake either through soil or foliar applied zinc fertilizers is explained (Fig 1).

The overall pathways for transport of zinc are quite complex (Fig 1). These pathways involve a variety of mechanisms to assimilate metals while preventing toxicity by regulation of transport, chelation, and sequestration [13]. Literature suggests the involvement of transporters such as members of the ZIP (zinc/iron-regulated protein) gene family, the cation diffusion facilitator (CDF) family, the P1B ATPase family etc. Besides these, the natural resistance-associated macrophage protein (NRAMP) family, and the Yellow Stripe1-Like (YSL) family are implicated in active metal homeostasis [14–17].

Although many gene families responsible for zinc transport are discovered the precise role of each of the protein at the molecular level is not understood yet. Further, the mechanisms underlying the transport of foliar applied zinc are not completely elucidated. The studies on the uptake of foliar applied zinc reported so far, are mostly limited to the visualization of zinc and its sub-cellular localization in the plant tissue. For e.g. zinc mobilization in a wheat plant using a stable isotope of zinc viz., ^65^Zn has been demonstrated [18]. In rice, using positron-emitting tracer imaging system (PETIS), zinc translocation in young leaves was confirmed [19]. To trace the presence of zinc in different parts of the wheat grain, the highly sophisticated micro-XRF technique was used [20]. Furthermore, at the molecular level, using functional complements of yeast mutants the genes responsible for metal uptake were identified [14]. In a study carried out in barley, soil application of ZnSO_4_ and its effect on the expression of plasma membrane metal transport family i.e.ZIP gene family in roots and shoots was reported [21]. However, such studies in case of foliar applied zinc are lacking. Since the aim of biofortification is to achieve high zinc concentration in wheat grain, a study of its accumulation in specific tissue that makes up the grain assumes importance. For example, accumulation of zinc in the gluten proteins might lead to variations in the end use property of wheat flour [22, 23]. A study carried out in wheat suggests that zinc application in the form of ^65^Zn resulted in zinc accumulation in glutenin fractions [24]. Yet another study reported increased levels of sulfur-rich (S-rich) gluten proteins upon zinc application in the form of ZnSO_4_.

The pathways in the transport of zinc were studied with zinc provided in its ionic form. In addition to ionic zinc, nanoparticulate zinc (especially zinc oxide nanoparticles) is being assessed as a source of zinc to achieve biofortification. In case of maize it was proved that when zinc oxide nanoparticles were applied as a foliar spray at low concentrations (50–200 mg/L) they were transported to the grain while at high concentrations (typically 1000 mg/L), the plants were unable to use the extra zinc and it was translocated to the soil through stem and roots [25]. These observations were made on the basis of quantification of zinc in plant parts and localization/visualization of particles under the transmission electron microscope (TEM).

In our laboratory, we have successfully developed a zinc complexed chitosan nanoparticles (Zn-CNP) formulation containing ~ 40 mg/L zinc. Scanning electron microscopy (SEM), zinquin staining of leaves and observation by confocal microscopy proved the uptake of Zn-CNP via stomata and zinc localization in leaves. Further staining of a section of the grain with dithizone and observation under a reflecting light microscope was a proof of enrichment of zinc in the endosperm, aleurone and embryo. The latter study revealed the association of zinc with endosperm proteins. To study whether Zn-CNP can be used as a foliar spray for biofortification, an experiment was conducted in durum wheat varieties with plants growing in sand supplemented with Hoagland’s medium devoid of zinc, instead of soil. In the experiment, one of the varieties (MACS 3125) was an indigenously developed high-yielding variety and the other was UC1114 (containing *Gpc-B1* gene responsible for high grain protein). Despite differences in the genotype, we observed 27 and 42% zinc enrichment in grain in MACS 3125 and UC1114, respectively [26]. Unlike earlier studies, we observed zinc enrichment when Zn-CNP containing 10 times lower zinc concentration was applied.

In line with our earlier work, the present study attempts at establishing a correlation between gene expression in flag leaf and grain zinc enrichment in plants grown in field conditions. We try to understand whether (i) the form in which the micronutrient is applied (Zn-CNP, 40 mg/L zinc, and ZnSO_4_, 400 mg/L zinc) causes a difference in the expression profiles of selected genes (ii) varietal differences [high yielding (MACS 3125) versus high protein containing (UC1114)] cause differential gene expression and (iii) the grain storage protein is altered/affected in response to Zn-CNP application.

## Materials and methods

### Plant growth conditions

Two durum wheat cultivars were used in the study viz., MACS 3125 (a high yielding variety developed by our institute and released for Maharashtra state). UC1114 (containing *Gpc-B1* transcription factor responsible for high grain protein, a kind gift from Prof. Jorge Dubcovsky, UC Davis, CA, USA). Seeds were grown in field conditions at the experimental farm at Hol, near Pune, India (18° 31’ N, 73° 55’ E) during the wheat growing season (November 2014 to March 2015).

### Zinc treatment

To know the basal levels of gene expression, different tissues such as flag leaves, lower leaves, stem, and spikes were collected at anthesis stage before plants received external zinc treatment. For time-dependent gene expression studies plants were grown separately, and 7 days after anthesis (DAA) foliar application of zinc complexed chitosan nanoparticles (Zn- CNP) was performed. Using a hand-sprayer, plants were sprayed till the entire leaf was covered. Three flag leaves were collected from zinc treated and untreated control plants at 2, 4, 8 and 12 h post-Zn-CNP treatment. For post anthesis expression studies zinc treatment involved (a) control, i.e. plants sprayed only with distilled water (no zinc application); (b) ZnSO_4_.7H_2_O (0.2%) and (c) Zn- CNP. Three flag leaves were collected from zinc treated and control plants each at an interval of 7 days beginning with seven days after anthesis (DAA) to 21 DAA.

### Zinc quantification

Zinc content in mature grains was determined in both cultivars (MACS 3125 and UC1114). Five plants were chosen randomly and wheat spikes were collected at maturity, and grains were harvested. Three representative grain samples (1 g each) were digested in three acid mixture consisting of nitric acid: sulphuric acid: perchloric acid in a ratio 3:2:1. Zinc quantification was performed using atomic absorption spectrophotometer (AAS, AAnalyst 400 Perkin Elmer, USA).

### Sampling and RNA extraction

Tissue samples were cut and flash frozen in liquid nitrogen and stored in -80°C until use. RNA was extracted using Plant RNA extraction kit (Spectrum, total RNA isolation kit, Sigma, USA) by following the manufacturer's instructions. RNA yield was quantified by recording the optical density (O.D.) at 260 nm using Nanodrop spectrophotometer (ND-1000, Thermo Fisher Scientific, USA). The integrity of extracted RNA was assessed by agarose gel (1%) electrophoresis using 0.5x Tris-borate EDTA buffer (TBE). Extracted RNA was treated with DNase I (Sigma, USA) to remove any residual DNA contamination.

### cDNA synthesis

cDNA was synthesized from 1μg of DNase-I-treated RNA using cDNA synthesis kit (Euroscript RT, Eurogentech, Belgium). Briefly, reaction mix, reverse transcriptase (RT) enzyme, oligo dT primers, RNA as a template and DEPC treated water was added to a vial in certain proportions as per manufacturer's instructions. The tube contents were then incubated at room temperature (27°C) for 10 min. Followed by incubation at 48°C for 60 min. The reaction was terminated by incubating at 95°C for 5 min, and the synthesized cDNA was stored at -20°C until use.

### qRT-PCR analysis

Genes for qPCR analysis were selected on the basis of their reported role in zinc uptake and transport. Genes selected were zinc (Zn)-regulated transporters and iron (Fe)-regulated transporter-like proteins i.e. ZIP gene family [27, 28]. Based on the literature, we selected 5 members (*ZIP1*, *ZIP3*, *ZIP7*, *ZIP10*, and *ZIP15*) of this family for our study, which are reported to show differential gene expression during the grain development stages [29]. In addition, genes encoding for the synthesis of enzymes involved in long-distance transport of zinc (phloem transporters *NAS* and *DMAS*), chloroplast exporter gene i.e. Heavy metal ATPase (*HMA*), the senescence-associated gene (*SAG*) and carbonic anhydrase (*CA*) gene producing the enzyme carbonic anhydrase which requires zinc as a cofactor were also selected.

Primers for each gene were obtained using sequences available at NCBI. Primer sequences for the target genes with amplicon size and accession number are summarized in Table 1. The chromosomal location of the gene was obtained from wheat expression database (http://wheat.pw.usda.gov/WheatExp/). The target gene expression was normalized using actin, a constitutive gene (GenBank accession no. (AB181991.1). All reactions were performed in triplicate, including non-template as the negative controls (NTC). qRT-PCR reactions included 2.5 μL diluted cDNA corresponding to 50 ng of total RNA, 2 μL SYBR Green PCR mix (Roche, Applied Science) 0.8 μL of both forward and reverse primers (final concentration 250 nM), and deionized water was used to make the reaction volume up to 10 μL. Gene expression was quantified using a capillary-based LC 2.0 real-time PCR system (Roche, Light Cycler 2.0, USA). PCR Cycling conditions used were 94°C—5 min, 45 cycles of 94°C—45 sec, 60°C ± 2°C—30 sec, 72°C—30 sec. Gene expression was studied using the comparative 2^-ΔΔCT^ method according to Livak and Schmittgen [30]. Dissociation curves were generated for each reaction to ensure specific amplification. Threshold values (C_T_) generated from the Software tool (Roche LC 2.0) were employed to quantify relative gene expression.

**Table 1:** Primers used for real-time PCR study.

Name	Sequence id/ Accession no.	Chromosome location	Sequence F: forward, R: reverse	Product Size (Base pair)
*Actin*	AB181991.1	1AL 1BL	**F** TTGTGCTCGACTCTGGTGAT **R** GCTCATAATCAAGGGCCACG	222
*CA*	AK331803.1	3AL	**F** CCGTCTGTGCTCTCAAGGTT **R** CATCGAAAGGCATTGAGGCG	185
*HMA2*	HM021132.1	7AL 7BL	**F** GGGCATCGTGTCAAACAGTT **R** CGCCAGTAAGCATCACTGAC	183
*ZIP 1*	DQ490133.1	3AL	**F** GTCCCCCTACTTCTACCGCT **R** TGGTTGACCCTCTGCCTGTT	237
*ZIP 3*	AY864924.1	2AL 2BL	**F** CACGCCCACTTGACAACATC **R** CTCCGGTGAGCTGACAACAA	116
*ZIP 7*	JQ690353.1	1AS 1BS	**F** GAGCTGATGCAACCACAAGG **R** GAGTGCGACACAATCCCCA	98
*ZIP 10*	AK334164.1	7AL 7BL	**F** GTGGATCTCATTGCTGCTGA **R** AGCCCAAATAGCCAGTGATG	123
*ZIP 15*	AK 333945.1	3AS	**F** CTCTCTGCGCTGGTTGCTTT **R** TGGGAGGACTCCGGCAACAG	132
*NAS2*	Traes_4BLBB0FC3BD3.2	4BL	**F** ATCGCACAGAAGTCCAAGGA **R** TGGCGAACTCCTCTCTCTTT	151
*DMAS1*	AB269908.1	4AS 4BL	**F** GATGGAGTACGTGGACCTGT **R** TTGCAGGAGAAATTGGCGAC	183
*SAG*	AK335587.1	1BS	**F** CAACAGCGACTTCTCCATCG **R** CGTCGATGAGCTTCAGGTTG	170

### Gluten quantification

Gluten protein extraction was performed using Osborne fractionation method followed by sequential extraction [31]. Each of the gliadin and glutenin extracts obtained was further separated on RP-HPLC (Waters, USA) by using C18 column (Poroshell 300SB, C18, 2.1mm ID × 75mm (5μm)) with a mobile phase of 1% trifluoroacetic acid (TFA). Acetonitrile gradient was set with a run time of 30 min. Protein absorption was measured at 210 nm using UV detector (2487 detector Waters, USA). The elution border of different protein type was set according to previous studies [32, 33].

### Statistical analysis

Comparison of means was performed by using analysis of variance (ANOVA). GraphPad Prism software (v 5.01) was used for performing the statistical analyses. Tukey’s multiple comparison test was used to compare means whenever ANOVA indicated significant differences among treatments. Pearson’s correlation analyses were carried out using two significance levels (P≤0.05 and 0.01).

## Results

### Grain zinc content

When the grain zinc content was estimated, it was observed that plants treated with zinc either in the form of Zn-CNP or ZnSO_4_ showed increased grain zinc in comparison with control (untreated plants) and the increase was statistically significant. The grain zinc content of UC1114 cultivar was higher than MACS 3125 cultivar (Table 2).

**Table 2:** Zinc content in whole seeds from two durum wheat cultivars.

Cultivars	Zinc Content (μg/g)		
Treatments	Control	Zn-CNP	ZnSO_4_ (0.2%)
MACS 3125	31.4±0.6	45.3 ±2.1***	53.1±2.8***
UC1114	38.2±1.3	48.1±2.0***	56.1±2.2***

Values are the averages of four independent biological replicate samples ± S.E.M. Two-Way ANOVA with Bonferroni's post-test (***P≤0.001).

### Tissue-specific ZIP (Plasma membrane transporters) gene expression at anthesis

Zinc is known to enter inside the cells via zinc-specific transporters present on the surface of plasma membrane. At first, the basal expression levels of the ZIP genes (viz., *ZIP1*, *ZIP3*, *ZIP7*, *ZIP10*, and *ZIP15*) in different tissues viz., flag leaves, non-flag leaves, stem and spike (S1 Fig) were estimated. The gene expression was studied without any external zinc treatment at anthesis stage (flowering stage) and data were normalized using ‘actin’ as housekeeping gene.

The expression pattern in other tissues such as non-flag leaves, stem, and spike showed variation as compared to flag leaves. The observed variation in different tissues at anthesis without external zinc application is shown in S1 Fig.

### Time-dependent gene expression at anthesis

qPCR data showed that *ZIP1*, *ZIP3*, and *ZIP7* were upregulated post foliar application of Zn-CNP (S2 Fig). Interestingly, it was observed that there was a switch between the expression levels of ZIP members at different time points. At 2 h and 4 h post foliar application of Zn-CNP, the levels of *ZIP1* transcript were 3–3.5 fold higher (S2A Fig). Expression levels of *ZIP3* and *ZIP7* at 2 and 4 h remained equal to untreated samples. With the increase in time, the levels of *ZIP3* increased significantly (>20 fold). Furthermore, *ZIP7* transcript levels were also elevated at 8 h post application (S2B and S2C Fig). It was observed that *ZIP10* expression levels were unchanged at all the time points and no significant differences were observed between untreated and Zn-CNP treated samples. However, *ZIP15* levels were found to increase by 2.5 to 3 fold at different time points as shown in S2D and S2E Fig.

It is reported that nutrient mobilization from leaves to grains is mainly carried out by flag leaves. The flag leaf is considered as a source of remobilized metals during senescence. Therefore, the effect of foliar zinc application (via Zn-CNP and conventional Zn-containing fertilizer) on the expression of metal homeostasis related genes was carried out in flag leaves at two-time points i.e.14 and 21 DAA. The transcript levels for the respective genes as determined by qPCR on 7 DAA were now used as a reference to monitor the time-dependent gene expression in both cultivars viz., MACS 3125 and UC1114.

### ZIP expression levels (plasma membrane transporters)

**Fig 2:**
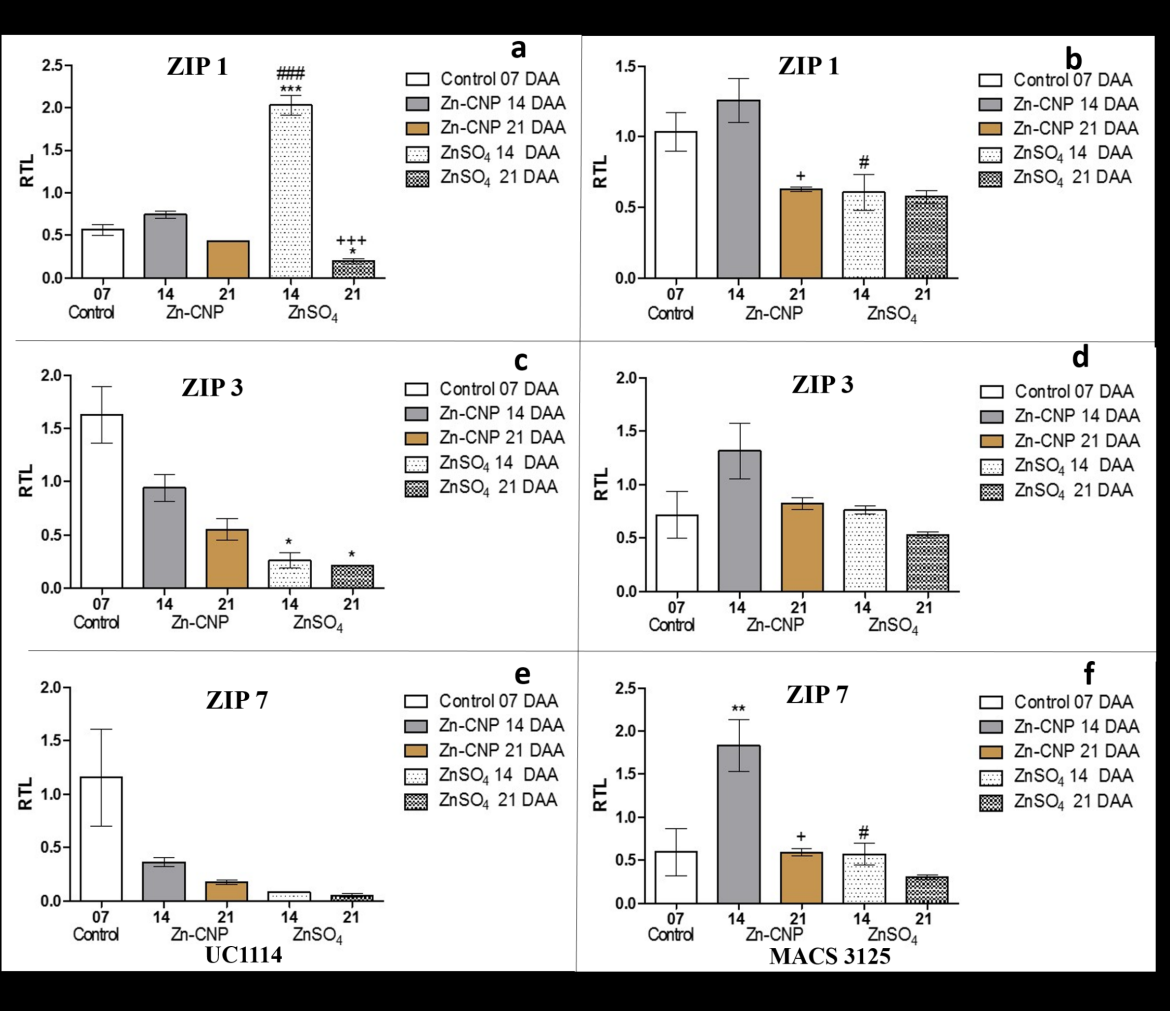
Relative expression levels of ZIP genes in flag leaves of two durum wheat cultivars collected at 7, 14 and 21 days after anthesis (DAA). a and b *ZIP1* UC1114 and MACS 3125 cultivar, respectively. c and d *ZIP3* UC1114 and MACS 3125 cultivar, respectively. e and f *ZIP7* UC1114 and MACS 3125 cultivar, respectively. Mean±S.E.M. (n = 3). One-Way ANOVA with Tukey’s multiple comparison test. *P<0.05,**P<0.01,***P<0.001 vs control, #P<0.05,##P<0.01,###P<0.001 Zn-CNP vs ZnSO_4_, +P<0.05,++P<0.01,P<0.001 14 DAA vs 21 DAA.

The transcript levels of *ZIP1* in UC1114 cultivar at 14 DAA were increased in flag leaves which received ZnSO_4_ treatment. This increase in case of UC1114 cultivar was significantly higher than untreated control at 7 DAA (P<0.001). However, at 21 DAA, *ZIP1* levels showed a decrease in both zinc treatments i.e. Zn-CNP and ZnSO_4_ (Fig 2A). In MACS 3125 cultivar, *ZIP1* levels in leaves receiving Zn-CNP were found to be similar to that of control at 14 DAA. However, with an increase in time after anthesis, the transcript levels were found to decrease (Fig 2B). Thus, in both cultivars (UC1114 and MACS 3125) a consistent decrease in the *ZIP1* transcript levels was observed in both zinc treatments at 21 DAA. Further, *ZIP3* levels in UC1114 cultivar also showed a decrease with an increase in time after anthesis. This decrease was statistically significant (P<0.05) in ZnSO_4_ treated samples at both 14 and 21 DAA (Fig 2C). A similar decrease in *ZIP3* level was not observed in MACS 3125 cultivar (Fig 2D). In the UC1114 cultivar, a decrease in the expression of *ZIP7* was observed with increase in time after anthesis in Zn-CNP and ZnSO_4_ treated samples at both 14 and 21 DAA (Fig 2E). While in case of MACS 3125 cultivar at 21 DAA the *ZIP7* levels showed decreased expression in both the zinc treatments (Fig 2F). Overall, the *ZIP1*, *ZIP3*, and *ZIP7* levels were decreased at 21 DAA in both zinc treatments as compared to control at 7 DAA.

**Fig 3:**
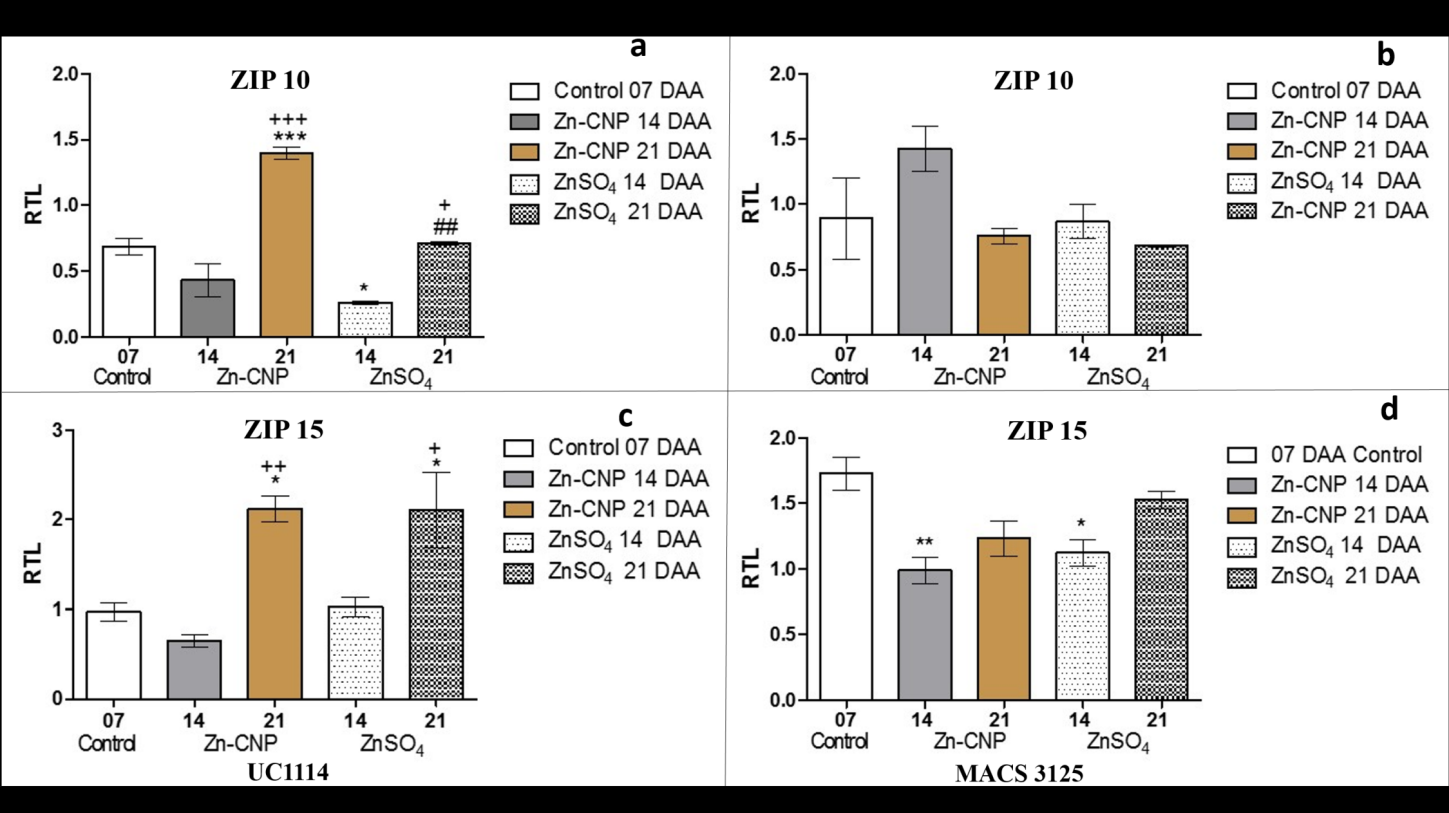
Relative expression levels (qRT-PCR, relative to actin expression) of ZIP genes in flag leaves of two durum wheat cultivars collected at 7, 14 and 21 days after anthesis (DAA). a and b *ZIP10* UC1114 and MACS 3125 cultivar, respectively. c and d *ZIP15* UC1114 and MACS 3125 cultivar, respectively. Mean±S.E.M. (n = 3). One-Way ANOVA with Tukey’s multiple comparison test. *P<0.05,**P<0.01,***P<0.001 vs control, #P<0.05,##P<0.01,###P<0.001 Zn-CNP vs ZnSO_4_, +P<0.05,++P<0.01,P<0.001 14 DAA vs 21 DAA.

Similarly, the expression levels of *ZIP10* and *ZIP15* was also monitored during grain development (Fig 3). In case of UC1114 cultivar, the transcript levels of *ZIP10* decreased at 14 DAA in both zinc treatments as compared to control at 7 DAA. However, at 21 DAA significant increase (P<0.05) in *ZIP10* transcript levels were observed in Zn-CNP as well as in ZnSO_4_ treated samples as compared to the levels at 14 DAA (Fig 3A). *ZIP10* transcript levels remained largely unaltered in both zinc treatments at 14 and 21 DAA in MACS 3125 cultivar,(Fig 3B). *ZIP15* transcript levels in UC1114 cultivar remained unchanged at 14 DAA in both zinc treatments (Zn-CNP and ZnSO_4_). However, as the time increased from 14 to 21 DAA, significant upregulation of *ZIP15* was observed in both zinc treatments (P<0.05) as shown in Fig 3C. In MACS 3125 cultivar, at 14 DAA a decrease in the gene expression of *ZIP15* was found in both Zn-CNP and ZnSO_4_ treated samples. At 21 DAA in both zinc treatments, a slight increase in the gene expression was observed which, however, was statistically not significant (Fig 3D). Thus the ZIP gene expression not only varied with an increase in time after anthesis but also with respect to the form in which the zinc was applied. Phylogenic analysis indicated that the members of the ZIP gene family (i.e. *ZIP1*, *ZIP3*, and *ZIP7*) are clustered together and hence responses of *ZIP1*, *ZIP3*, and *ZIP7* to external zinc treatment are probably similar. The *ZIP10* and *ZIP15* formed a different clade all together and hence showed a variable response (S3 Fig).

### Long distance transport (Phloem transporters)

**Fig 4:**
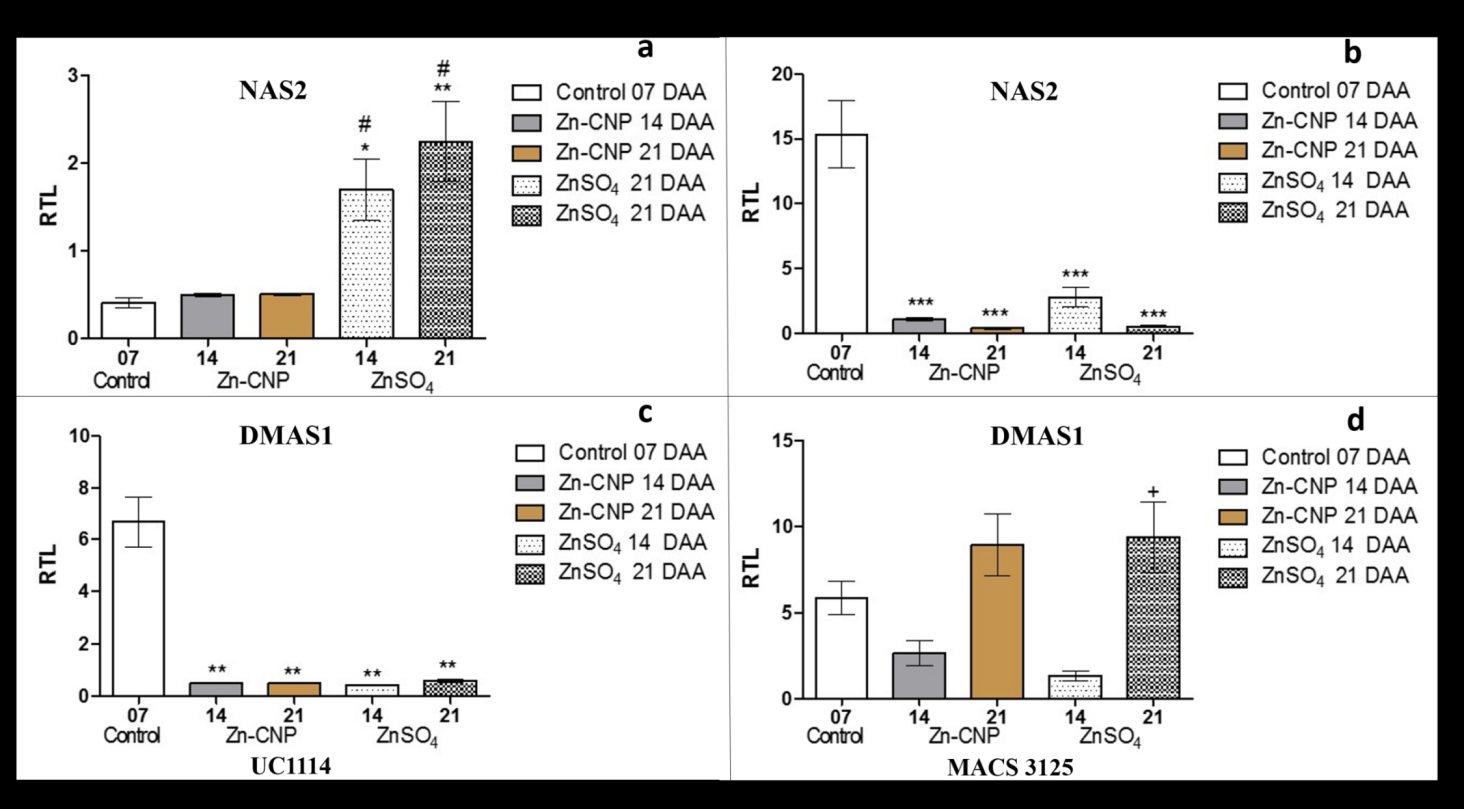
Relative expression levels of phloem transporters in flag leaves of two durum wheat cultivars collected at 7, 14 and 21 days after anthesis (DAA). a and b *NAS2* UC1114 and MACS 3125 cultivar, respectively. c and d *DMAS1* UC1114 and MACS 3125 cultivar, respectively. Mean±S.E.M. (n = 3). One-Way ANOVA with Tukey’s multiple comparison test. *P<0.05,**P<0.01,***P<0.001 vs control, #P<0.05,##P<0.01,###P<0.001 Zn-CNP vs ZnSO_4_, +P<0.05,++P<0.01,P<0.001 14 DAA vs 21 DAA.

The phloem represents the main route by which nutrients are translocated from flag leaves into grains. It was observed that zinc application in the form of Zn-CNP or ZnSO_4_ resulted in significant changes in the expression levels of nicotianamine synthase (viz., *NAS2*). In UC1114 cultivar, *NAS2* transcript levels were significantly up-regulated (P<0.05) at both 14 and 21 DAA in ZnSO_4_ treated samples, whereas a similar increase was not observed in Zn- CNP treated samples. The observed trend could be attributed to the higher amount of zinc (10 times higher) applied in the form of ZnSO_4_. In MACS 3125 cultivar, a similar response was not observed (Fig 4B).

Besides *NAS2* gene, we have studied the expression levels of *DMAS1*. DMA synthase (*DMAS*) converts NA to DMA. In UC1114 cultivar, the *DMAS1* expression significantly decreased (P<0.01) in both zinc treatments at 14 and 21 DAA (Fig 4C). On the other hand, in MACS 3125 cultivar, *DMAS1* transcript levels in Zn-CNP, as well as ZnSO4, treated samples showed a statistically significant increase (P<0.05) at 21 DAA in comparison with the 14-day time-point (Fig 4D).

### Effect of zinc on expression levels of other genes

**Fig 5:**
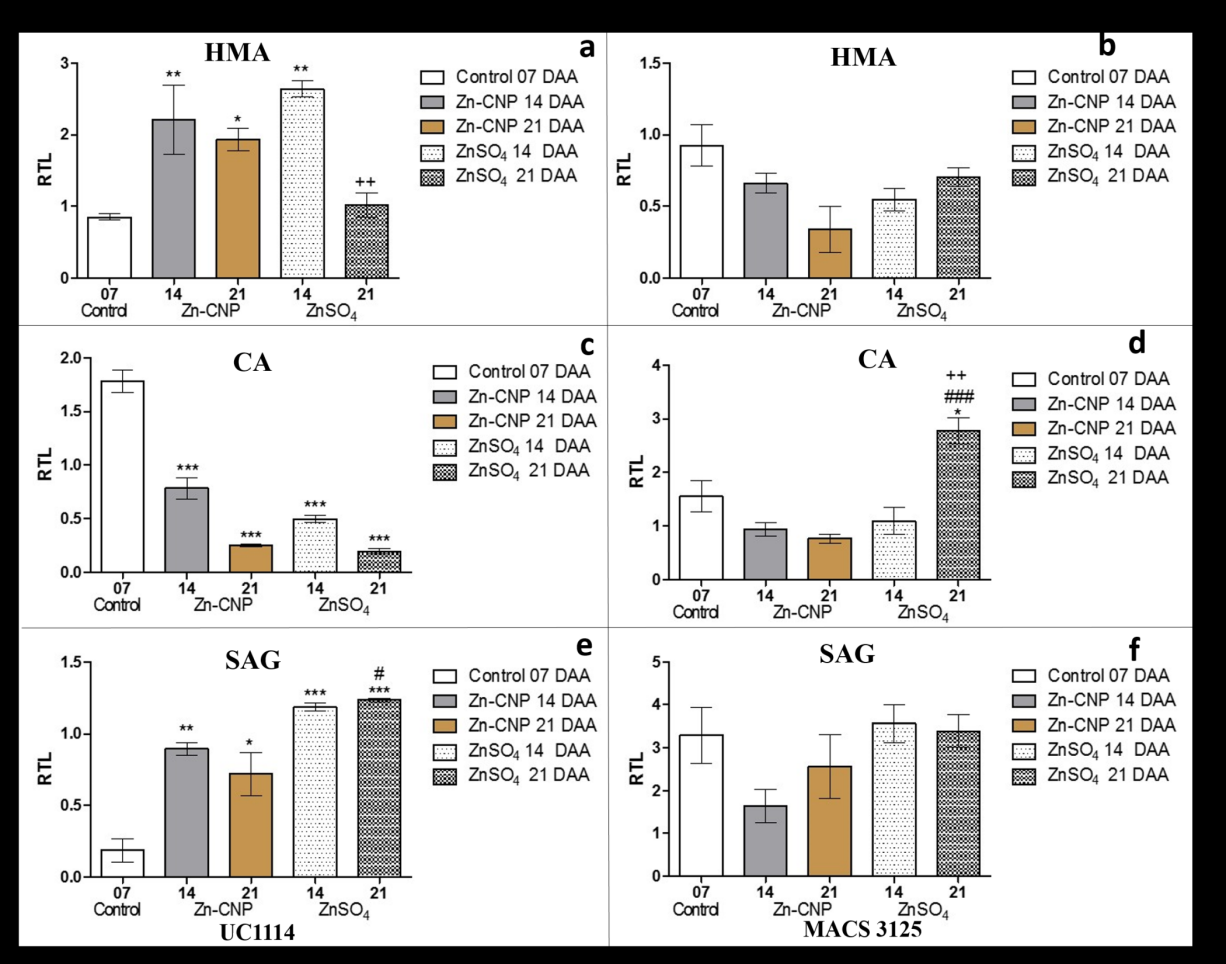
Relative expression levels of other genes in flag leaves of two durum wheat cultivars collected at 7, 14 and 21 days after anthesis (DAA). a and b *HMA* UC1114 and MACS 3125 cultivar, respectively. c and d *CA* UC1114 and MACS 3125 cultivar, respectively. e and f *SAG* UC1114 and MACS 3125 cultivar, respectively. Mean±S.E.M. (n = 3). One-Way ANOVA with Tukey’s multiple comparison test. *P<0.05,**P<0.01,***P<0.001 vs control, #P<0.05,##P<0.01,###P<0.001 Zn-CNP vs ZnSO_4_, +P<0.05,++P<0.01,P<0.001 14 DAA vs 21 DAA.

During the investigation on the changes in gene expression in the flag leaf tissue in response to foliar zinc application, the transcript levels of few other genes involved in zinc homeostasis were also examined. Genes studied were heavy metal ATPase (*HMA*), carbonic anhydrase and senescence-associated genes (*SAG*). HMA expression in UC1114 cultivar decreased with increase in time after anthesis i.e. from 7 to 21 DAA. At 14 and 21 DAA, significant down-regulation of HMA levels was observed in both Zn-CNP, and ZnSO_4_ treated samples (Fig 5A). A similar trend was observed in MACS 3125 cultivar (Fig 5B). Irrespective of the form in which zinc was applied, the trends in the expression of the transcript remained unchanged.

The effect of foliar zinc application on the transcript levels of carbonic anhydrase (*CA*) was also studied (Fig 5C and 5D). Zinc acts as a cofactor for the enzyme and its expression depends on the zinc concentration in the plants. In UC1114 cultivar, *CA* transcript levels were significantly increased (P<0.05) at 14 DAA in both cultivars receiving zinc treatment i.e. Zn-CNP and ZnSO_4_. However, as compared to 14 DAA, the *CA* transcript levels at 21 DAA were lower in both zinc treatments (Fig 5C). In MACS 3125 cultivar, CA levels were higher at 21 DAA in ZnSO_4_ treated samples while in Zn-CNP treated samples CA levels remained unaffected (Fig 5D).

### Senescence-associated gene expression

The effect of zinc on the senescence-associated gene (*SAG*), transcript levels were estimated at 7, 14 and 21 DAA (Fig 5E and 5F). In UC1114 cultivar (Fig 5E), at 14 and 21 DAA, both Zn-CNP and ZnSO_4_ treatment resulted in significant up-regulation of *SAG* gene (P<0.05). However, in MACS 3125 cultivar, a similar increase in the expression of *SAG* was not observed in both zinc treatments, and the levels were comparable to control at 7 DAA (Fig 5F). In addition, the *Gpc-B1* gene expression in UC1114 cultivar was also studied. *Gpc-B1* mRNA levels increased with application of Zn-CNP and ZnSO_4_ (S4 Fig). To further confirm the onset of senescence chlorophyll content of the flag leaves at 7, 14 and 21 DAA were also measured (S5 Fig). With the increase in time after anthesis decrease in chlorophyll content was observed in all the treatments i.e. control, Zn-CNP, and ZnSO_4_.

**Fig 6:**
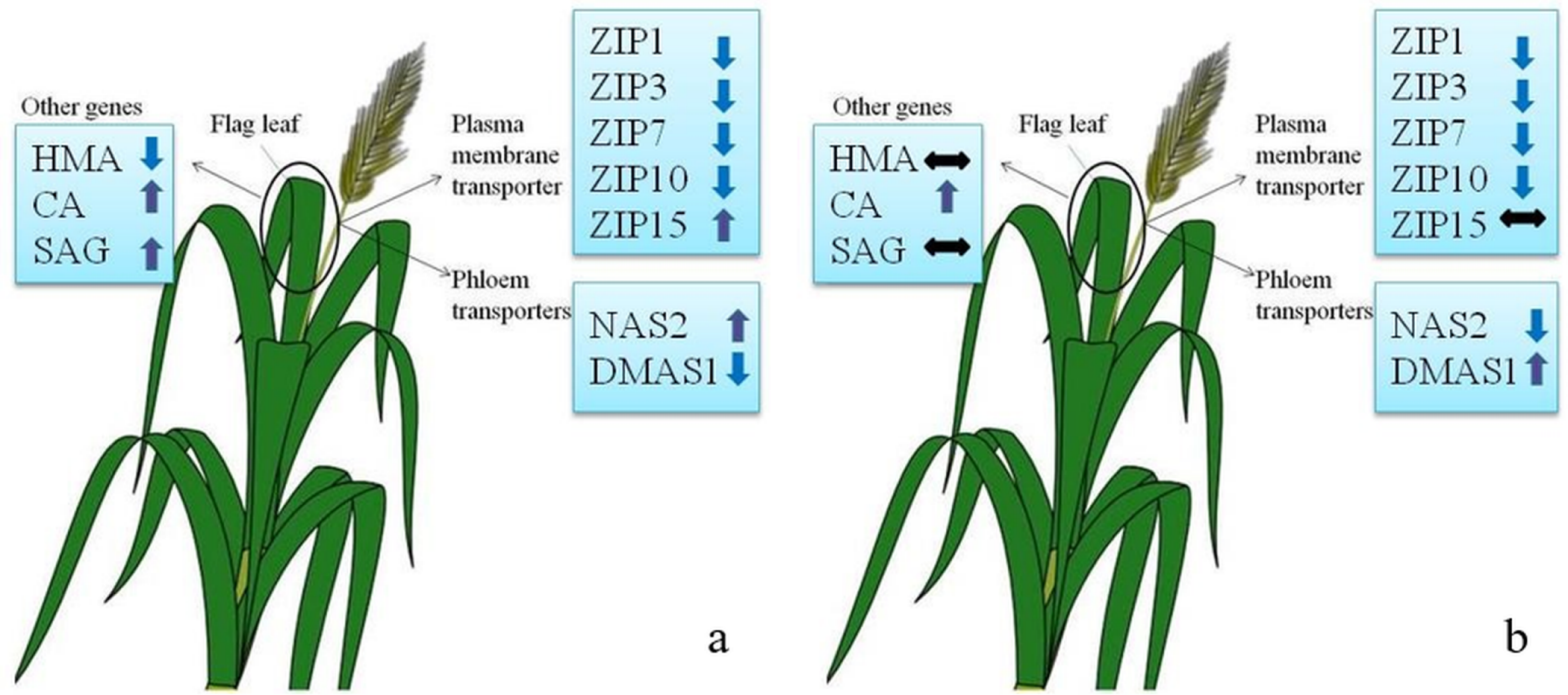
Schematic showing Zn-CNP dependent overall gene expression pattern across two durum wheat cultivars at 21 days after anthesis (DAA). a: UC11114 and b: MACS 3125.

The overall gene expression across two durum wheat cultivars i.e. UC1114 and MACS 3125 is shown in Fig 6. Amongst different genes studied, the overall response at 21 DAA for ZIP gene family members was similar in both the cultivars, whereas for other genes, the expression pattern varied across the cultivars.

### Correlation between grain zinc content and gene expression in flag leaves

To elucidate the relationships between gene expressions in flag leaves at 14 and 21 DAA with zinc content in mature grains, Pearson’s correlation analyses were performed (Table 3). Correlation analysis between zinc content and transcript levels of selected genes could give an idea about the involvement of the metal transporter genes in the grain zinc loading. In UC1114 cultivar, seed zinc content was negatively correlated with *ZIP1*, *ZIP3*, *ZIP7*, *HMA* and *CA* genes and positively correlated with *Gpc-B1* gene. Further, in MACS 3125 a similar trend was observed. Flag leaf expression was negatively correlated with *ZIP1*, *ZIP3* at both14 and 21 DAA, whereas a positive correlation was observed for *ZIP15* and *CA* genes at 21 DAA (Table 3).

**Table 3:** Correlation analysis between metal transport-related genes in flag leaves during grain development (14 and 21 DAA) and zinc content in grains in UC1114 and MACS 3125 cultivar.

Gene	UC1114		MACS 3125	
	14 DAA	21 DAA	14 DAA	21 DAA
*ZIP1*	-0.9695	-0.9880*	-0.9695	-0.9880*
*ZIP3*	-0.9319	-0.9855	-0.9739	-0.9849
*ZIP7*	0.3461	-1.000***	0.2610	-0.08498
*ZIP15*	-0.6025	0.9326	-0.8702	0.9970*
*HMA*	-0.9817	-0.9404	-0.9648	0.1625
*CA*	-0.9184	-0.9797	0.9622	0.9094
*Gpc-B1*	0.9131	0.9980*	NA	NA

NA: Not available (*P<0.05, ***P<0.001)

### Effect of zinc fertilization on gliadins and glutenins

**Fig 7:**
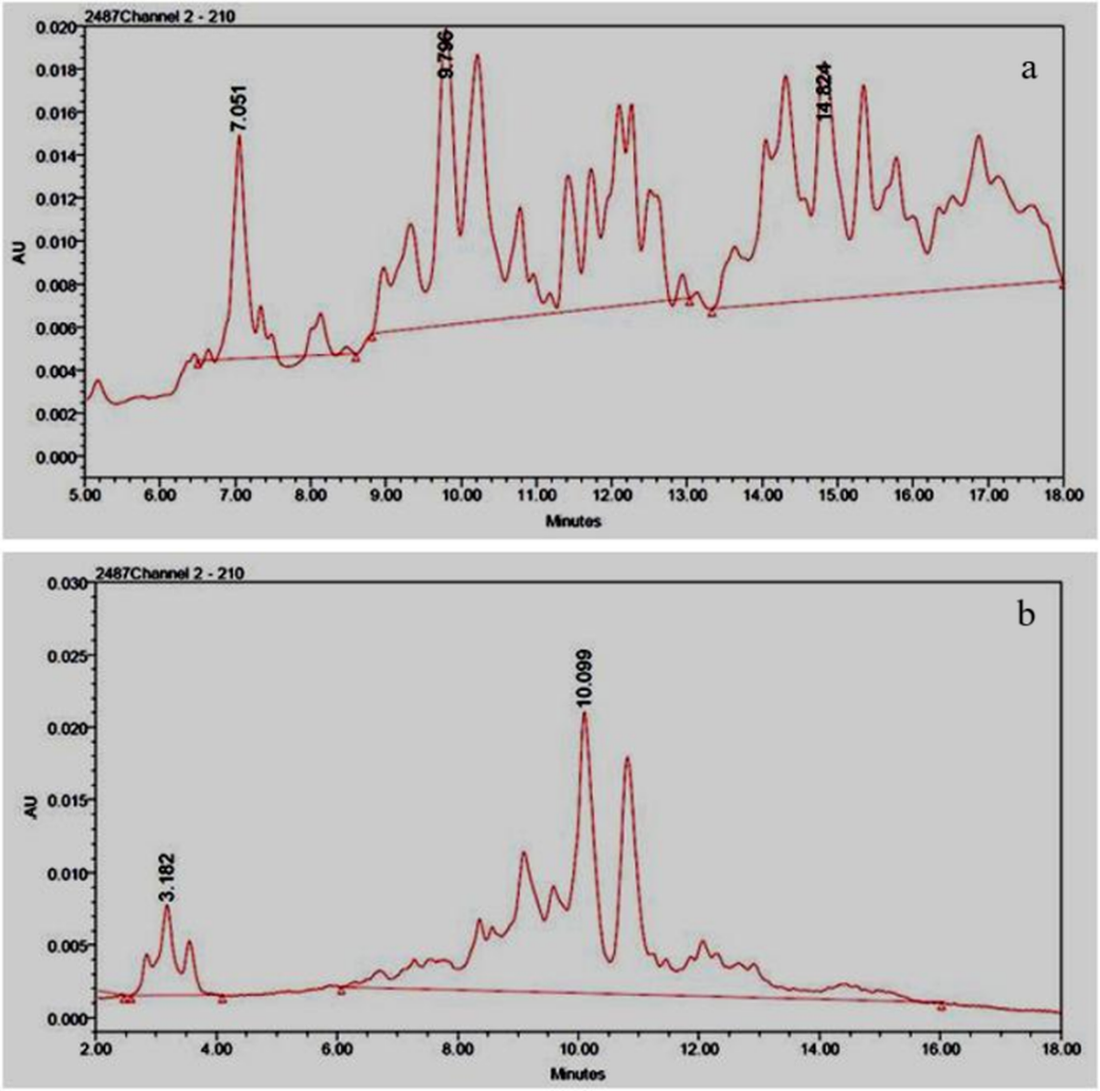
Representative chromatograms depicting separation of gluten proteins using RP-HPLC. a: different subunits of gliadins, b: high molecular weight (HMW) and low molecular weight (LMW) glutenins.

The endosperm consists of two types of proteins i.e. storage proteins and structural proteins. Structural proteins in wheat are also known as gluten proteins. To further understand the changes in the composition of proteins in response to enriched zinc the gliadin and glutenins were separated into individual subtypes using RP-HPLC. The chromatograms for the separation of gluten proteins are shown in Fig 7. Gliadin subunits were eluted from the C-18 column on the basis of hydrophobicity of the proteins, first, ω-gliadins, followed by α and β- gliadins and finally S-rich γ-gliadins. The retention time (Rt) for these gliadin subunits was 7.0, 9.7 and 14.8 min, respectively (Fig 7A). Glutenins were separated as high molecular weight glutenins (HMW, Rt 3.1 min.) and low molecular weight glutenins (LMW, Rt 10.0 min (Fig 7B). The relative quantities of different protein types were derived from the area under the curve in the chromatograms.

**Fig 8:**
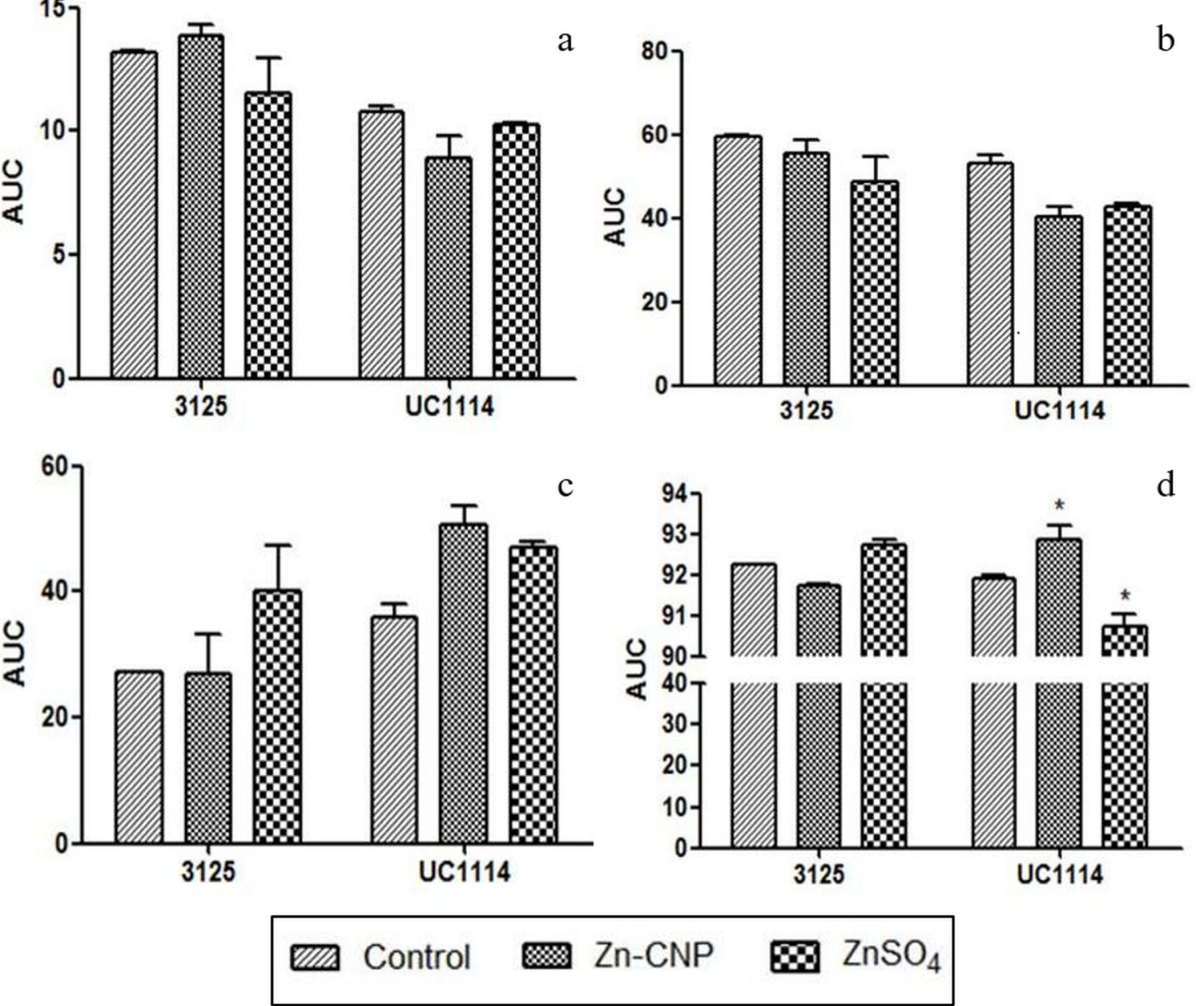
Quantification of gluten proteins. a: ω-gliadins, b: α and β gliadins, c: γ-gliadins and d: Low molecular weight (LMW) glutenins, Mean±SEM (n = 3). *P<0.05.

In the case of MACS 3125 and UC1114 cultivars, zinc treatments (Zn-CNP and ZnSO_4_) led to no significant variations in the S-poor ω gliadin and α and β gliadins content in the two cultivars (Fig 8A and 8B). However, changes in S-rich γ gliadins were evident upon zinc treatment. In MACS 3125 cultivar highest γ gliadins were found in grain samples which received ZnSO_4_ treatment followed by those who received Zn-CNP treatment. In UC1114 cultivar increase in γ type of gliadin was observed in both treatments i.e. Zn-CNP and ZnSO_4_ (Fig 8C). Quantitatively, the percent increase in γ -type gliadins were slightly higher than that of ω, α, and β gliadins. Our study also revealed that gliadin content in grain endosperm also depends on the type of cultivar studied. In UC1114 cultivar total gliadin content was always higher than MACS 3125 cultivar. Foliar application of Zn-CNP and ZnSO_4_ resulted in increased levels of low molecular weight (LMW) glutenins (Fig 8D).

## Discussion

Foliar zinc application during grain development stages showed significant grain zinc enhancement. Data obtained in the present study shows similarity with earlier findings [11, 12]. The application of Zn-CNP showed significant grain zinc enhancement at a low dosing regimen which could be attributed to the sustained release of zinc from the nanocarrier as well as high residence time on leaves. This finding proves that despite the contribution of zinc through the soil, foliar applied Zn-CNP brings about grain zinc enhancement. The variation obtained in grain zinc content of two cultivars could be attributed to the presence of *Gpc-B1* a QTL in UC1114 cultivar which is responsible for enhanced protein and zinc content [34].

Our results are similar to an earlier study [25] where the application of zinc oxide nanoparticles (ZnO) in maize plant increased the zinc content. However, in present study ionic zinc is complexed with chitosan nanoparticles. The zinc provided in the form of Zn-CNP is plant ‘bio-available’ and hence improvement in grain zinc content was realized. The uptake of zinc from foliar applied ZnSO4 is assisted by cuticular pores or hydration pores on the leaf surface. While in case of Zn-CNP, uptake takes place through stomata owing to the large diameter of stomatal openings. Once internalized, Zn-CNP release zinc ions due to acidic intercellular pH and further zinc uptake and transport depends on the transporters present on the surface of the plasma membrane [26].

Several members of the Zn-regulated transporters in the iron (Fe) -regulated transporter-like (IRT) protein (ZIP) gene family have been characterized and shown to be involved in metal uptake and transport in plants [29]. Time-dependent increased expression in the ZIP members studied, shows the specificity of individual ZIP members towards the zinc (S2 Fig).

Further, the observed differences in the transcript levels for two different zinc treatments could also be attributed to the difference in the available zinc; a 10-fold difference in the zinc concentration of Zn-CNP and ZnSO4. The variation in the levels of ZIP genes across different time points of grain development from 7 to 21 DAA indicates stringency in regulation amongst ZIP members. The levels also varied across cultivars/genotypes signifying the response of external zinc application to two different cultivars. Our results are in agreement with a study in barley plant which showed that zinc deficiency induced up-regulation of several ZIP members and subsequent application of zinc in the form of ZnSO4, resulted in decreased expression of ZIP members [21]. It is already known that ZIP expression is regulated by several factors viz. zinc availability in surrounding environment; pH and type of tissue [35–37]. Yeast mutants expressing *A*. *thaliana* ZIP genes revealed that zinc uptake depends on variation in the K*m* and V*max* values which indicate binding affinity and the rate of zinc transport, respectively [38]. Similarly, expression of ZIP was also found to be induced by zinc deficiency while some ZIP members were constitutively expressed to regulate the zinc ions [39]. The results showing temporal variations observed in the expression of ZIP members at different developmental stages in the present study corroborate well with previous data.

Besides plasma membrane zinc transporters, the expression of phloem zinc transporters was also assessed. Phloem is the major pathway for translocation of nutrients from leaves to grains. It is reported that zinc ions exhibit low solubility in the alkaline phloem sap, hence they are transported by forming a complex with metal ion chelators [40]. Nicotinamine (NA) is the major chelator involved in phloem transport of zinc and iron. Nicotinamine synthesis is regulated by nicotianamine synthase (*NAS*) gene which converts its precursor *S*-adenosyl methionine to NAS. In the present study, *NAS2* expression levels in both cultivars (MACS 3125 and UC1114) showed a significant variation and this difference could be due to the involvement of multiple genes in the regulation of *NAS* gene. Recently 23 new members of NAS gene family were identified in wheat [41].

The NA is converted into deoxy-mugineic acid via an intermediate step involving two enzymes i.e. nicotianamine aminotransferase (NAAT) and deoxy-mugineic acid synthase (*DMAS*). In our study, we observed variation in the expression level of *DMAS1* gene across cultivars in both zinc treatments. According to earlier studies, variable responses in *DMAS* expression were reported in different plant species. Transcriptome analysis of wheat leaves during senescence did not show altered levels of *DMAS1* [29]. *DMAS1* was reported to be involved in translocation of iron compounds in rice [42]. Furthermore, under zinc deficiency, *DMAS* expression was significantly increased in roots but not seen in the shoots [19]. The observed variability in the response of *NAS2* and *DMAS1* transcript levels in the present study indicates the possible involvement of different mechanisms of long-distance zinc transport across cultivars.

The effect of external zinc application on the expression levels of *HMA*, *CA* and *SAG* were performed. The *HMA* transporters in leaf tissue are located on chloroplasts and their physiological role is to export the zinc ions from the chloroplast to cytoplasm. These metal ions are later on translocated to developing grains. In our study, we found the decreased levels of *HMA* transcripts during grain development stage from 7 to 21 DAA. The decreased level of these exporters may be attributed to the changes in the intracellular levels of zinc as well as the form in which zinc was applied (i.e. Zn-CNP or ZnSO4). Results obtained in the present study are similar to other studies which report up-regulation of *HMA* in endosperm tissue at 25 DAA with no considerable increase in leaves [43].

The results on *HMA* expression in flag leaves are not in agreement with the changes in the transcriptome analysis of leaves as reported by Pearce et al. [29]. The possible explanation for the difference observed in the present study might be correlated to the role of *HMA* (export of zinc from chloroplast) during senescence. Since the plants receiving foliar zinc treatment have higher zinc levels, the chloroplast export seems to be energetically unfavorable. Further, expression level of carbonic anhydrase (*CA*) was also influenced by zinc application. Zinc acts as a cofactor for the enzyme activity of *CA*. Overall, the results obtained in the present study resemble earlier studies which demonstrate that the intracellular levels of zinc have a positive correlation to carbonic anhydrase gene transcript and its enzyme activity [44, 45].

A good correlation between senescence and nutrient translocation to grains has been observed [46]. Enhanced expression of *SAG* gene in UC1114 cultivar could possibly be due to the presence of *Gpc-B1*, a transcription factor responsible for grain protein enhancement and associated accelerated senescence [47]. This was confirmed by qPCR analysis of *Gpc-B1* gene which showed significant up-regulation in UC1114 cultivar (S4 Fig). Most of the translocated nutrients originate mainly from degradation of chloroplast proteins during leaf senescence. It has been demonstrated that *TaSAG12* transcript levels are upregulated during leaf senescence [48]. The decrease in chlorophyll content with increase in time after anthesis further confirms the observed *SAG* transcript up-regulation and the onset of senescence (S5 Fig). In light of the results obtained in the present study (i.e. upregulation of *Gpc-B1* and *SAG* genes with an increase in time after anthesis) the possible mechanism responsible for more efficient zinc translocation is being reiterated.

Applying foliar zinc during early stages of reproductive development could be an effective way of increasing seed zinc concentration [49]. It may be noted that changes in mRNA levels at 14 and 21 DAA may not correlate with changes in protein or enzyme activity, due to post-transcriptional processing. On the other hand, the expression profiles revealed in this study could be a starting point for detailed studies on the molecular mechanisms underlying the transport of foliar applied zinc to seeds.

In addition to the gene expression analysis, the effect of foliar Zn-CNP on grain endosperm protein was also evaluated. Our earlier study has shown that zinc enrichment occurs in embryo, aleurone, and endosperm but the concentration in each of these parts can vary [26]. Therefore, in the present study grain endosperm proteins were quantified post zinc enrichment. In wheat, major endosperm proteins are known as gluten proteins which are further divided into gliadins (monomeric) and glutenins (polymeric) which form inter- and intra-chain disulfide bridges [31]. Increased S-containing gluten proteins upon both zinc treatment could be attributed to the role of zinc which acts as a global transcriptional regulator and cofactor. The activity of several enzymes such as protein disulfide isomerase, nitrate reductase (NR) and glutamine synthetase (GS) increase after zinc fertilization. [22, 50]. Therefore, it is possible that zinc is responsible for induction/regulation of several genes that could result in increased gliadin content as observed in the present finding. A study in barley also suggests that zinc fertilization increases the expression of hordein genes and in turn affect the protein composition [23]. Similarly, in the present study, we propose that zinc fertilization affects the transcription levels of several genes involved in gluten synthesis. However, a separate investigation is needed to confirm these findings.

Furthermore, a significant correlation has been observed between the concentration of grain zinc and the protein content [51]. It is reported that zinc treatment results in increased levels of S-rich gliadins and zinc forms tetrahedral complexes with S containing amino acids, cysteine, and histidine [52, 53]. Our results are in agreement with above studies in which zinc fertilization has increased the S-rich γ gliadin subunits.

Based on the gene expression data, we propose the molecular mechanism underlying the zinc uptake post foliar application of Zn-CNP. The ZIP gene expression pattern in both cultivars across 14 and 21 DAA showed a similar response in Zn-CNP and ZnSO4 treated samples. A further study of more number of the genes in the family would give a better trend. Taken together this result suggests that the uptake of zinc via Zn-CNP at the molecular level is analogous to the uptake of zinc from ZnSO4.

## Conclusion

With recent advancements in technology, the demand for use of nanomaterials for the delivery of agrochemicals is increasing. At this stage, efforts towards understanding the plant-nanoparticles interaction are extremely important. Due to lack of studies on the underlying interactions, several concerns on the use of nanomaterials have been indicated, which can only be resolved experimentally. Our earlier study using Zn-CNP showed its utility as a nanocarrier suitable for biofortification. In this study, we have proved that foliar application of Zn-CNP influences expression of several genes implicated in the transport of zinc. Transport of micronutrients is a complex phenomenon and data obtained indicates that genes with increased expression during grain filling may be essential for metal translocation to seed. The gene expression pattern of samples under Zn-CNP treatment (containing 10 times lower dose of zinc) was comparable with ZnSO_4_ treated samples proving the efficacy of nanocarrier-mediated zinc enhancement without adverse effects on the plant metabolism. Our study also shows the effect of zinc enrichment on grain storage proteins particularly the gluten fraction. In our opinion, such studies can improve our understanding of the processes underlying nanocarrier-mediated delivery of agrochemicals.

## Supporting information

S1 Fig Tissue-specific relative gene expression in ZIP gene family members. a: *ZIP1*, b: *ZIP3*, c: *ZIP7*, d: *ZIP10* and e: *ZIP15*. Mean±S.E.M., (n = 3).
(TIF)

S2 Fig Time-dependent gene expression in ZIP gene family members. a: *ZIP1*, b: *ZIP3*, c: *ZIP7*, d: *ZIP10* and e: *ZIP15*. Mean±S.E.M., (n = 3).
(TIF)

S3 Fig A Phylogenetic tree for the ZIP gene family members. Amino acids from putative transporters within each family were aligned using CLUSTAL W and a neighbor-joining tree constructed using pairwise deletions and 1,000 bootstrap iterations with the program MEGA 5.
(TIF)

S4 Fig Relative expression levels of *Gpc-B1* gene in flag leaves of UC1114 cultivar collected at 7, 14 and 21 days after anthesis (DAA). Mean±S.E.M. (n = 3). One-Way ANOVA with Tukey’s multiple comparison test. *P<0.05,***P<0.001 vs control, +P<0.05,++P<0.01 14 DAA vs 21 DAA.
(TIF)

S5 Fig Chlorophyll content of wheat leaves during senescence measured by Arnon’s method [54]. Mean±S.E.M., (n = 3).
(TIF)

## Abbreviations

AAS: atomic absorption spectrophotometry
CA: carbonic anhydrase
CDF: cation diffusion facilitator
DAA: days after anthesis
DMAS: deoxy-mugineic acid synthase
Fe: Iron
HMA: heavy metal ATPase
HMW glutenins: high molecular weight glutenins
LMW glutenins: Low molecular weight glutenins
NAS: nicotianamine synthase
NRAMP: natural resistance associated macrophage protein
S: sulphur
S.E.M: Standard error of mean
qPCR: quantitative polymerase chain reaction
RP-HPLC: reverse phase high performance liquid chromatography
SAG: senescence-associated gene
YSL: yellow stripe like family
Zn: Zinc
ZIP: zinc/iron-regulated protein family
ZnO: zinc oxide nanoparticles
ZnSO_4_: zinc sulphate
Zn-CNP: zinc loaded chitosan nanoparticles
