# Supplementary figures and images for: Nanocarrier-mediated foliar zinc fertilization influences expression of metal homeostasis related genes in flag leaves and enhances gluten content in durum wheat
Source: PLoS One. 2018 Jan 17;13(1):e0191035. doi: 10.1371/journal.pone.0191035 (PMC5771588; doi:10.1371/journal.pone.0191035)

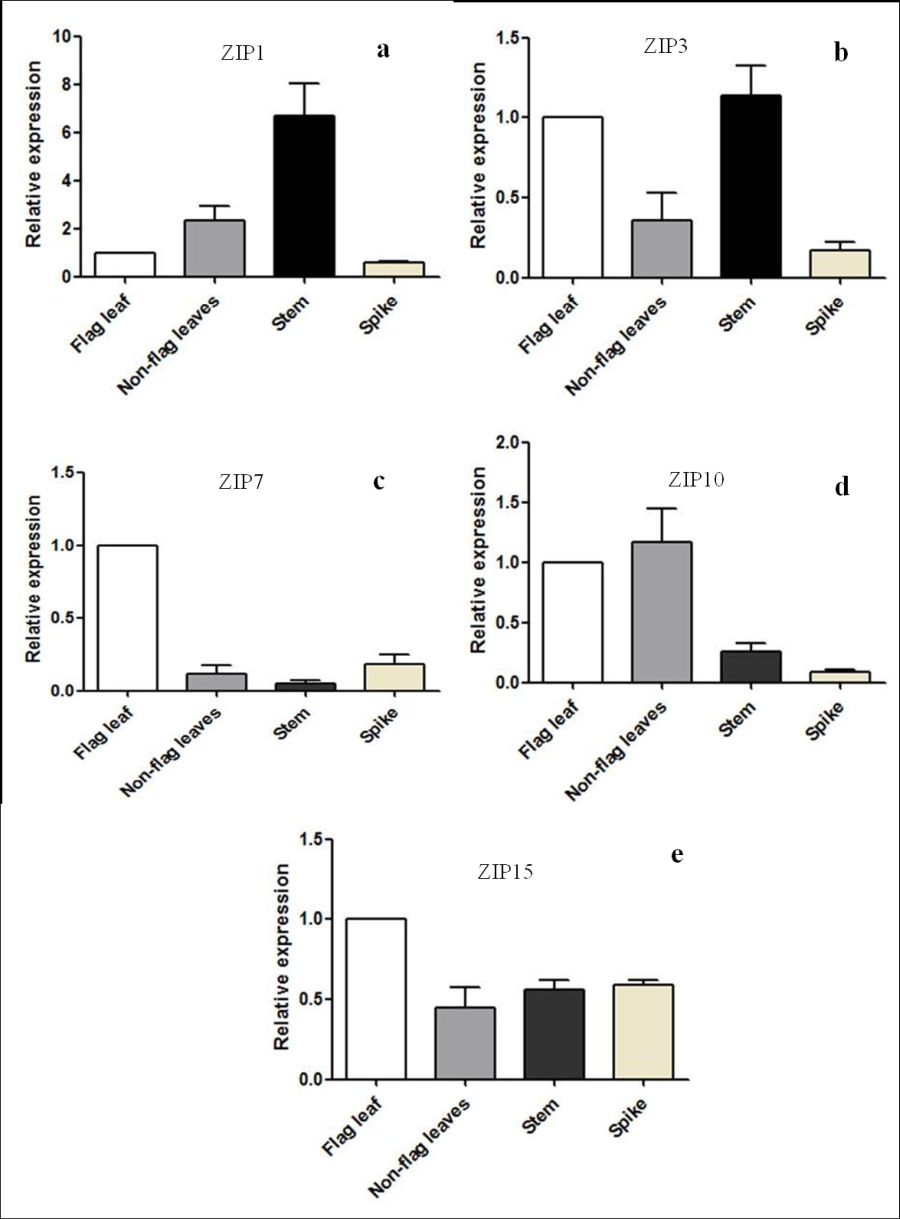

Supplement: S1 Fig — a: ZIP1, b: ZIP3, c: ZIP7, d: ZIP10 and e: ZIP15. Mean±S.E.M., (n = 3). (TIF) [file pone.0191035.s001.tif]

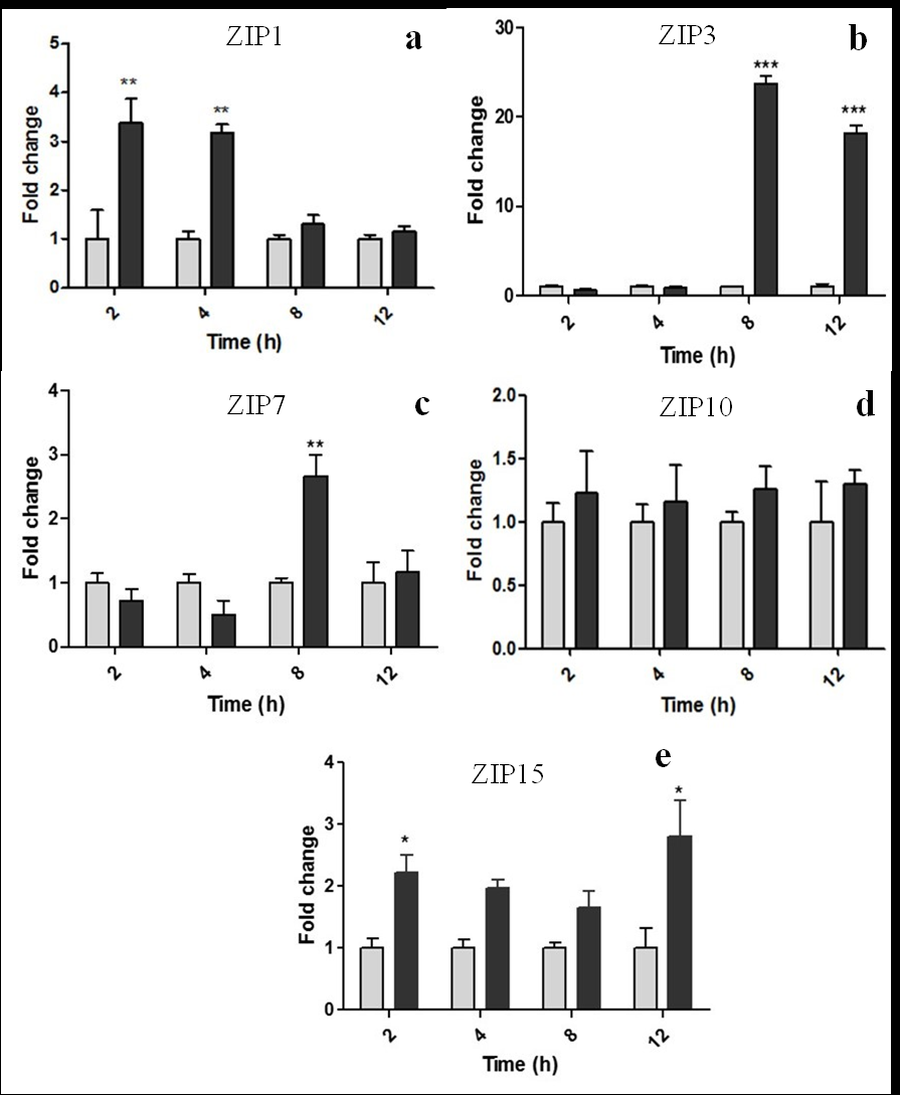

Supplement: S2 Fig — a: ZIP1, b: ZIP3, c: ZIP7, d: ZIP10 and e: ZIP15. Mean±S.E.M., (n = 3). (TIF) [file pone.0191035.s002.tif]

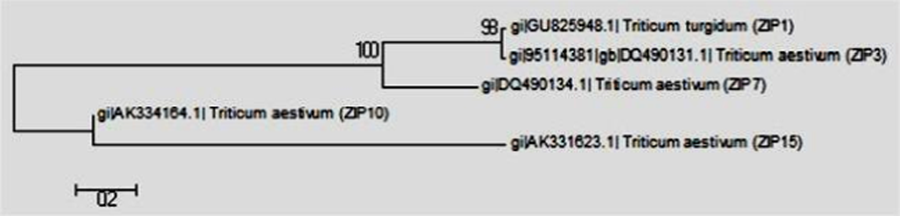

Supplement: S3 Fig — Amino acids from putative transporters within each family were aligned using CLUSTAL W and a neighbor-joining tree constructed using pairwise deletions and 1,000 bootstrap iterations with the program MEGA 5. (TIF) [file pone.0191035.s003.tif]

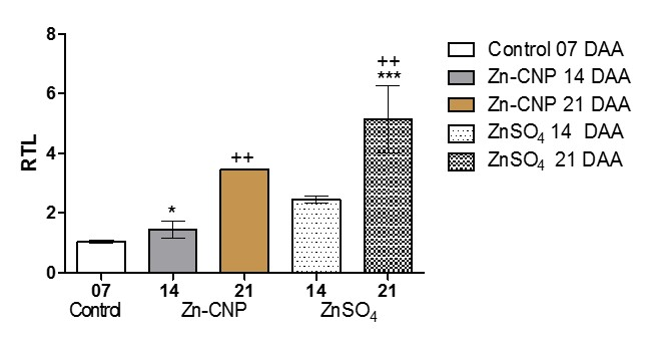

Supplement: S4 Fig — Mean±S.E.M. (n = 3). One-Way ANOVA with Tukey’s multiple comparison test. *P<0.05,***P<0.001 vs control, +P<0.05,++P<0.01 14 DAA vs 21 DAA. (TIF) [file pone.0191035.s004.tif]

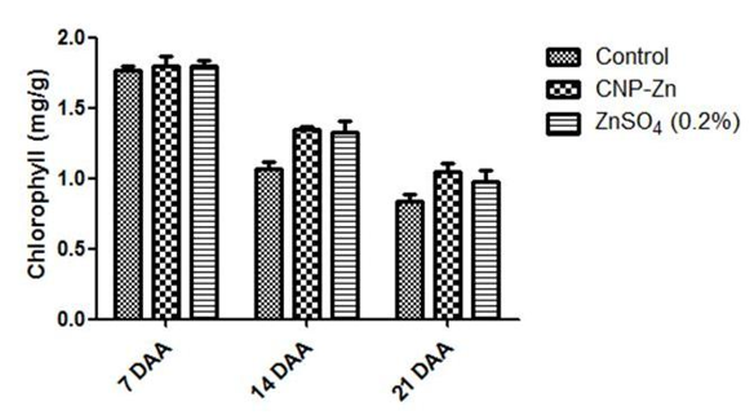

Supplement: S5 Fig — Mean±S.E.M., (n = 3). (TIF) [file pone.0191035.s005.tif]
